# Supplementary figures and images for: Generation of a HiBiT-expressing recombinant rat hepacivirus supporting both in vivo and in vitro infection
Source: PLoS Pathog. 2026 Apr 8;22(4):e1014127. doi: 10.1371/journal.ppat.1014127 (PMC13086425; doi:10.1371/journal.ppat.1014127)

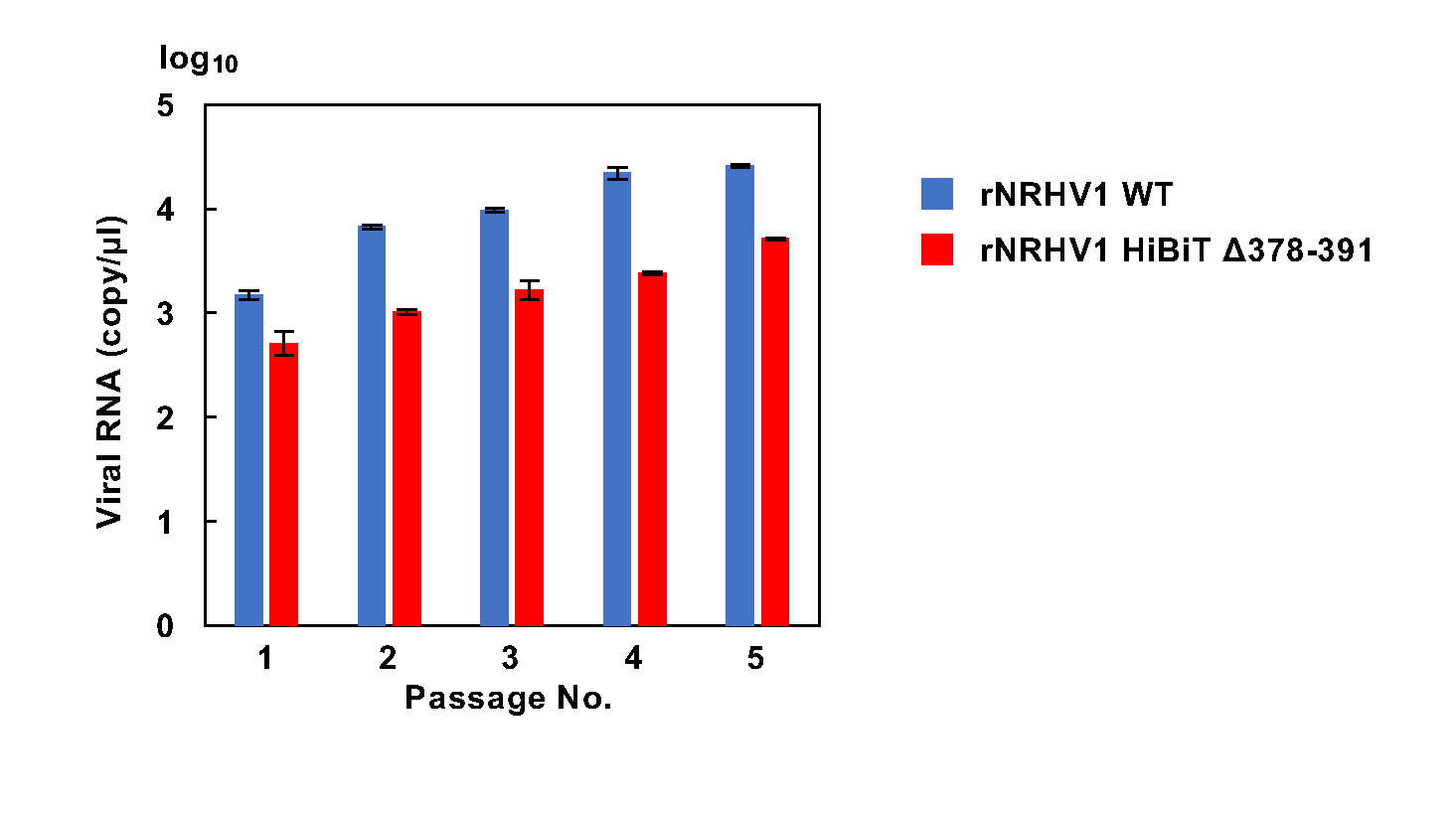

Supplement: S1 Fig — Culture supernatants collected from cells transfected with CPER products at 6 days post-transfection (dpt) were designated as passage 0 (P0). P0 supernatant was inoculated into naïve McA1.8 cells, and the resulting supernatant collected at 96 hours post-infection (hpi) was designated as P1. This passaging procedure was repeated sequentially to generate supernatants designated as P2 through P5. Viral RNA copy numbers in the supernatants were quantified by qRT-PCR. (TIF) [file ppat.1014127.s001.tif]

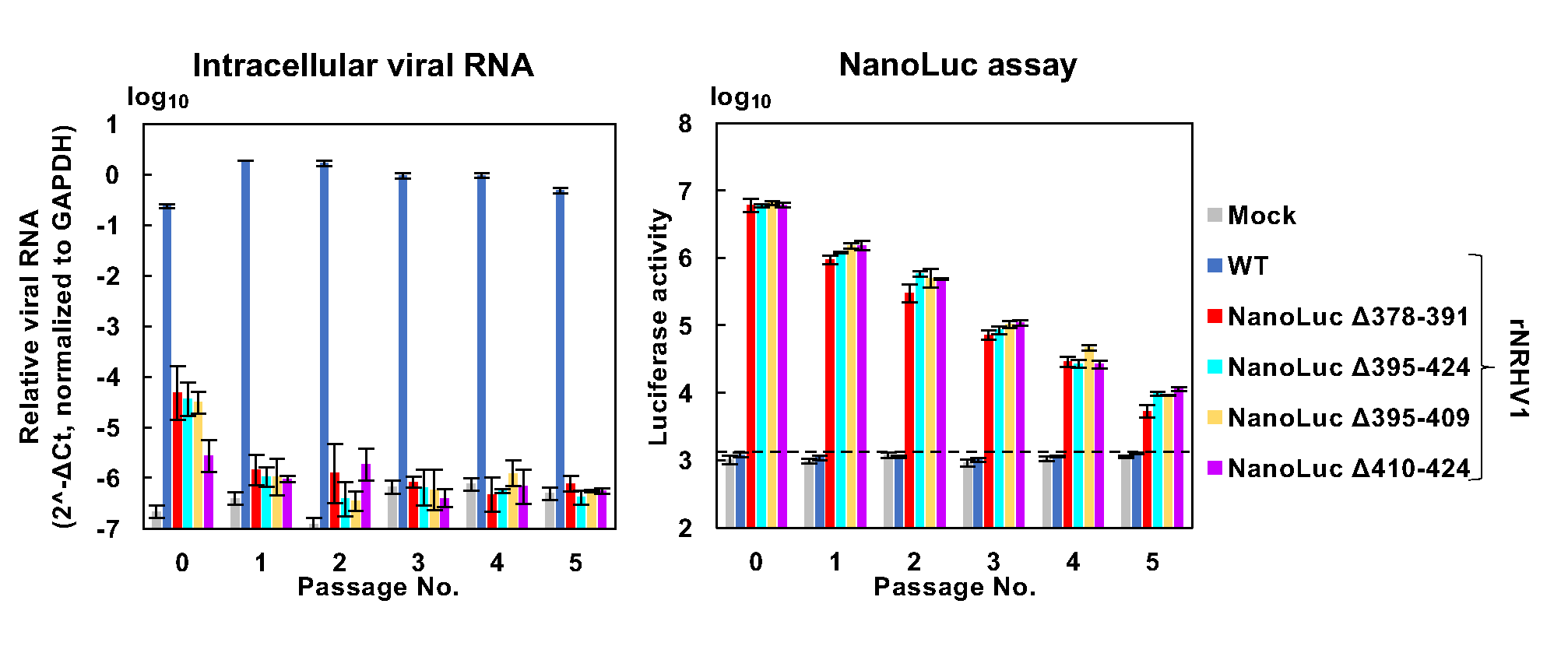

Supplement: S2 Fig — Supernatants were collected from cells transfected with CPER-derived viral preparations at 6 dpt and designated as passage 0 (P0). P0 supernatants were then inoculated into naïve McA1.8 cells, and the resulting supernatants collected at 96 hpi were designated as P1. This passaging procedure was repeated sequentially to generate P2 through P5. Subsequently, 100 µl of each passage supernatant (P0–P5) was inoculated into naïve McA1.8 cells. Intracellular NRHV1 RNA levels and luciferase activity were assessed at 96 hpi. Intracellular NRHV1 RNA levels were normalized to GAPDH and calculated as 2^-ΔCt. (TIF) [file ppat.1014127.s002.tif]

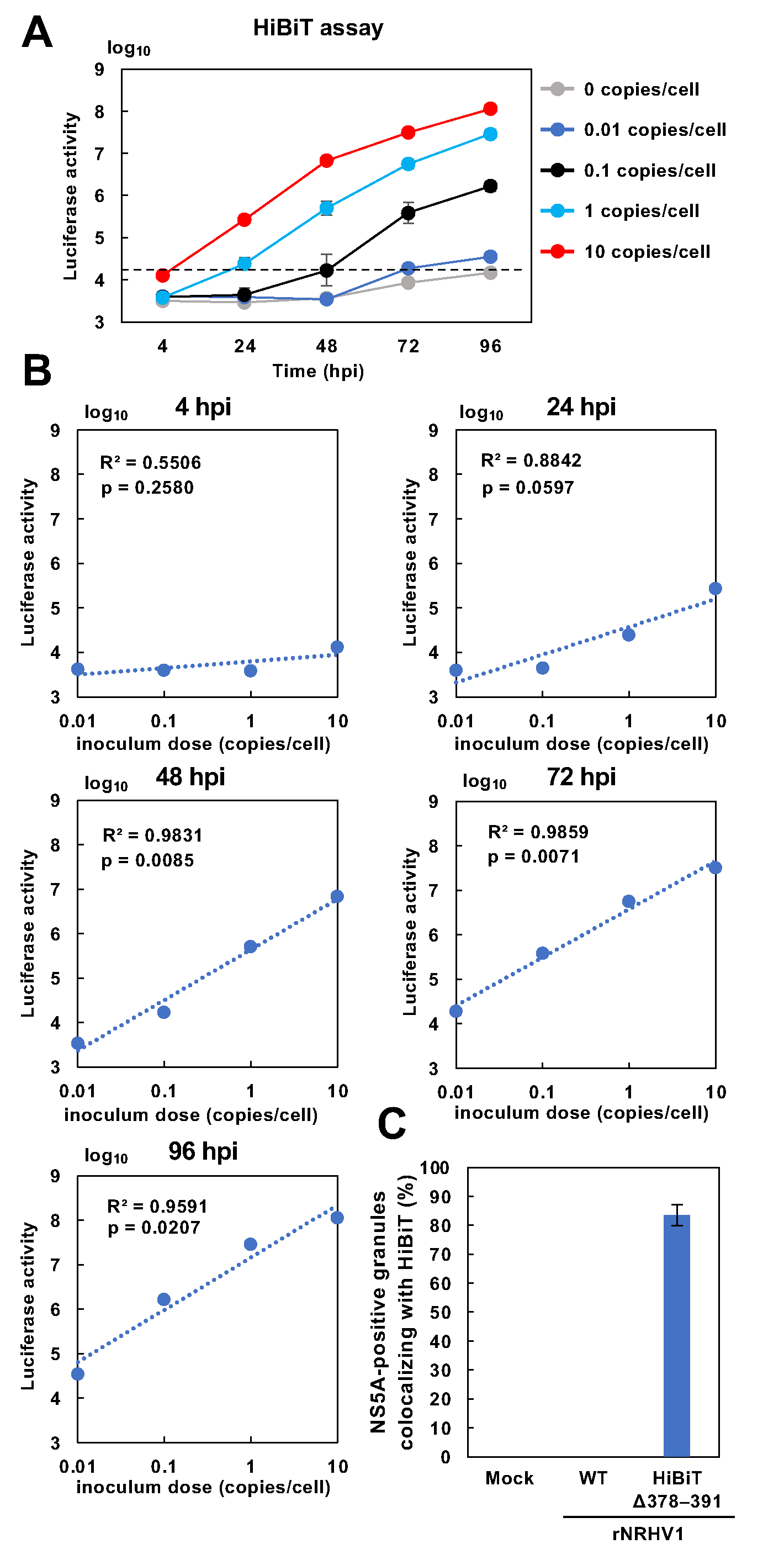

Supplement: S3 Fig — (A) McA1.8 cells were infected with rNRHV1 HiBiT Δ378–391 at 0, 0.01, 0.1, 1, or 10 copies/cell. Luciferase activity was measured at the indicated time points. (B) Correlation between rNRHV1 HiBiT Δ378–391 inoculum (copies/cell) and luciferase activity at each indicated time point. Scatter plots were generated from the data shown in (A). Linear regression analysis was performed on log–log transformed data, and the coefficient of determination (R²) was calculated for each time point. P-values were calculated from the slope of the regression line using a t-test. (C) Colocalization between NS5A and HiBiT signal in immunofluorescence images was quantified using ImageJ. Thresholding was applied uniformly to all images within each experiment to generate binary masks. Granules were defined using particle analysis after size filtering to exclude background noise. Overlapping granules were identified using the Image Calculator (AND function). The percentage of NS5A-positive granules overlapping with HiBiT-positive granules was calculated for each field of view (n = 5 independent fields). (TIF) [file ppat.1014127.s003.tif]

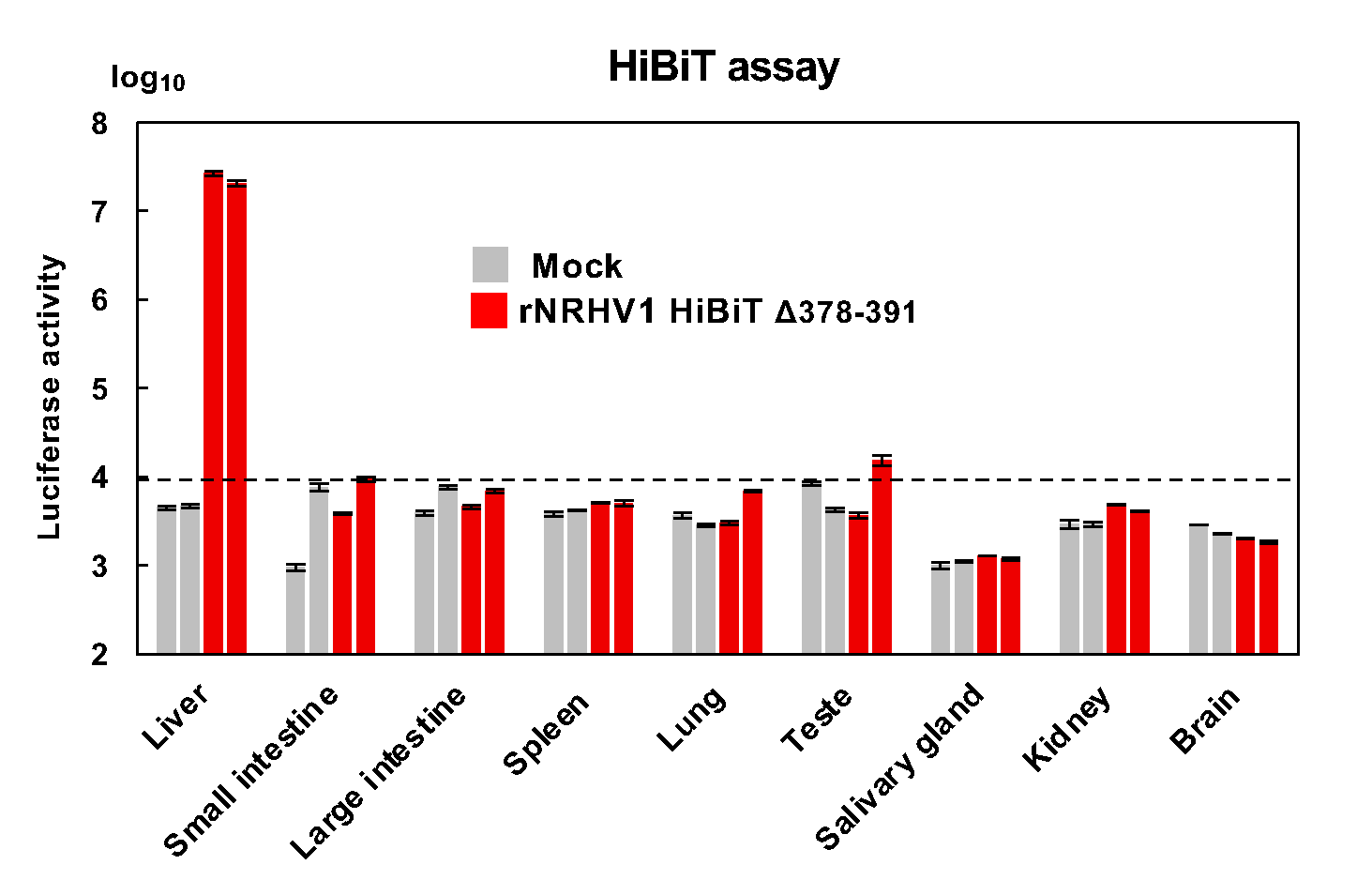

Supplement: S4 Fig — HiBiT activity in 10 µg of tissue lysate was measured. Individual organs were collected from rNRHV1 HiBiT Δ378–391–infected mice (mouse No. 1 and No. 3) and from a mock-infected control mouse. (TIF) [file ppat.1014127.s004.tif]

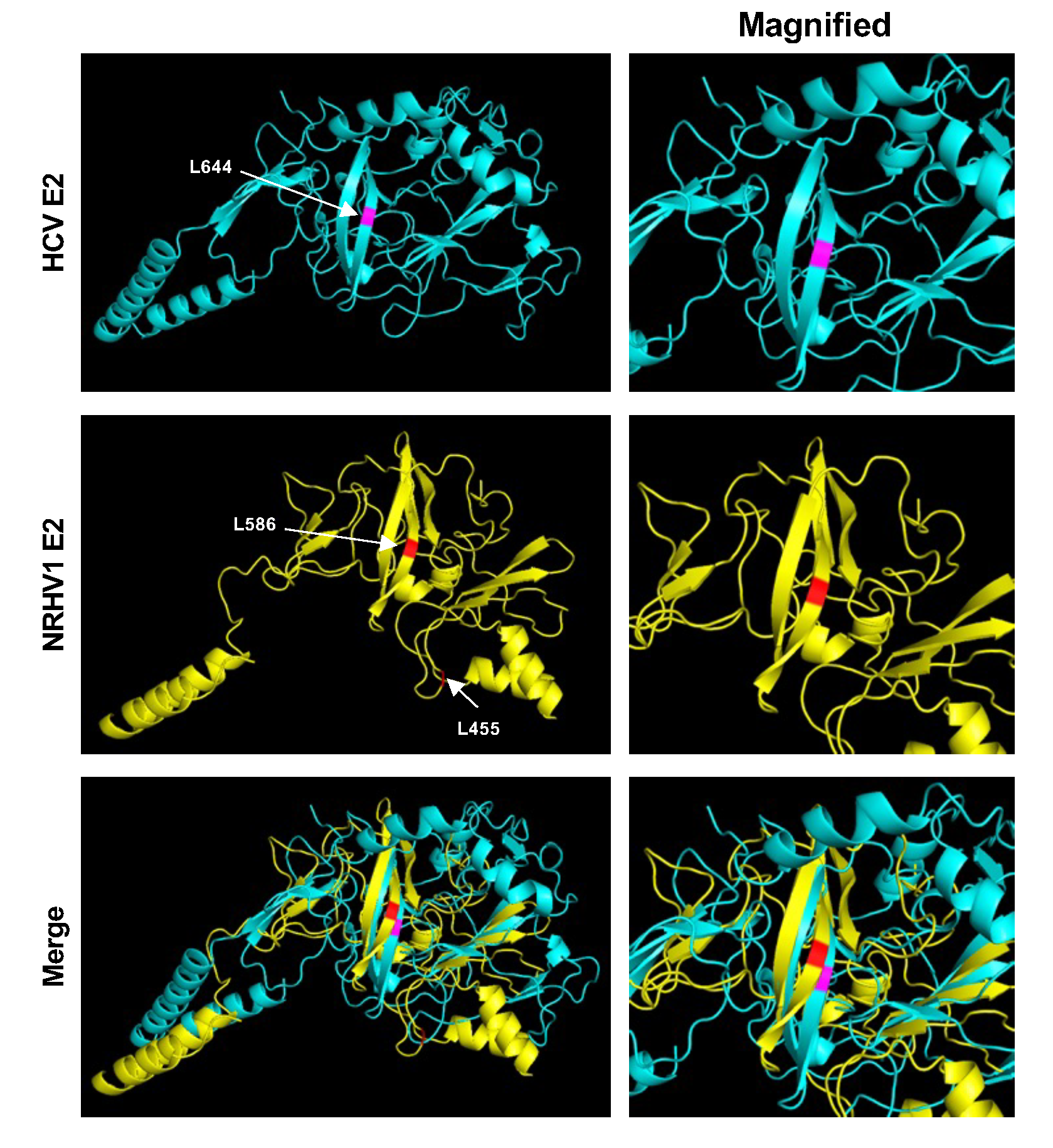

Supplement: S5 Fig — The 3D structures of HCV E2 (blue) and NRHV1 E2 (yellow) were predicted using AlphaFold2. (TIF) [file ppat.1014127.s005.tif]

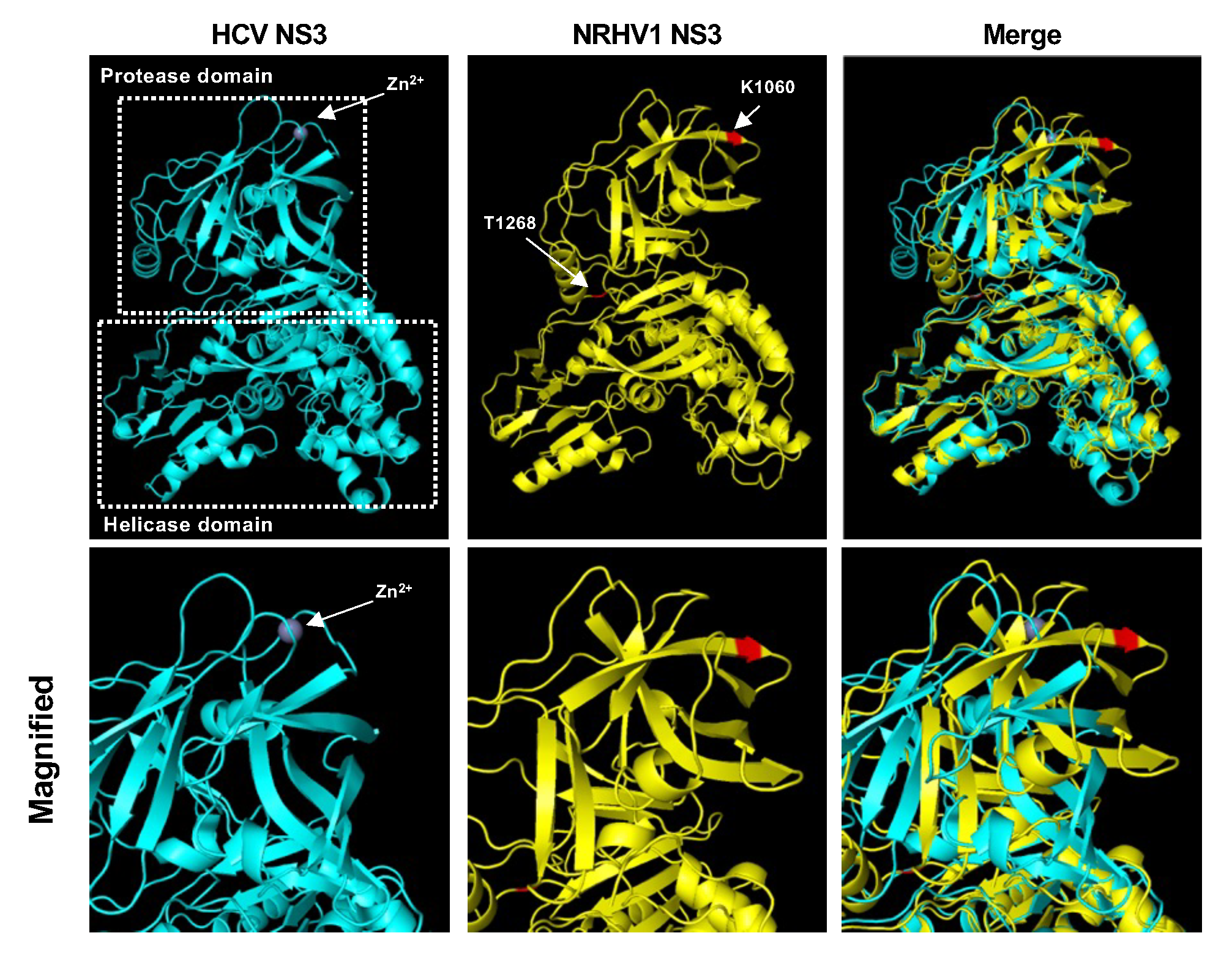

Supplement: S6 Fig — Ribbon diagrams show the crystal structure of HCV NS3 (PDB ID: 3O8B, blue) and the predicted structure of NRHV1 NS3 (yellow). (TIF) [file ppat.1014127.s006.tif]
